# Effect of Pulmonary Inflammation by Surface Functionalization of Zinc Oxide Nanoparticles

**DOI:** 10.3390/toxics9120336

**Published:** 2021-12-06

**Authors:** Ayoung Jung, Sung-Hyun Kim, Jun-Young Yang, Jayoung Jeong, Jong Kwon Lee, Jae-Ho Oh, Jin Hee Lee

**Affiliations:** Division of Toxicological Research, National Institute of Food and Drug Safety Evaluation, Ministry of Food and Drug Safety, Osong, Cheongju 28159, Korea; kntp2002@korea.kr (A.J.); tjdgus32@korea.kr (S.-H.K.); yangjy@korea.kr (J.-Y.Y.); 0jjy@korea.kr (J.J.); jkleest@korea.kr (J.K.L.); chopin68@korea.kr (J.-H.O.)

**Keywords:** nanoparticles, zinc oxide, intratracheal instillation, bronchoalveolar lavage, acute inflammation

## Abstract

Zinc oxide nanoparticles (ZnO NPs) are used in various industries such as food additives, cosmetics, and biomedical applications. In this study, we evaluated lung damage over time by three types of ZnO NPs (L-serine, citrate, and pristine) following the regulation of functional groups after a single intratracheal instillation to rats. The three types of ZnO NPs showed an acute inflammatory reaction with increased LDH and inflammatory cell infiltration in the alveoli 24 h after administration. Especially in treatment with L-serine, citrate ZnO NPs showed higher acute granulocytic inflammation and total protein induction than the pristine ZnO NPs at 24 h. The acute inflammatory reaction of the lungs recovered on day 30 with bronchoalveolar fibrosis. The concentrations of IL-4, 6, TNF-α, and eotaxin in the bronchoalveolar lavage fluid (BALF) decreased over time, and the levels of these inflammation indicators are consistent with the following inflammatory cell data and acute lung inflammation by ZnO NP. This study suggests that single inhalation exposure to functionalized ZnO NPs may cause acute lung injury with granulocytic inflammation. Although it can recover 30 days after exposure, acute pulmonary inflammation in surface functionalization means that additional studies of exposure limits are needed to protect the workers that produce it.

## 1. Introduction

In recent years, as the number of cases of applying nanomaterials in various industrial fields has increased, it has become a critical issue to identify the toxicity of nanomaterials accurately. Primarily, zinc oxide nanoparticles (ZnO NPs) are used in many commercial products, including food additives, cosmetics, textiles, paints, and personal hygiene products [1]. In addition, ZnO NPs are widely used as an ingredient of paints and coating and finishing materials in products and buildings because they provide long-term protection from ultraviolet light [2,3].

The increasing use of ZnO NPs has raised concerns about their potential toxicity to humans and the environment. ZnO NPs might enter the human body by various routes, including oral ingestion, nasal inhalation, intravenous injection, and transdermal delivery [4,5,6,7,8]. Therefore, the toxicological properties of ZnO NPs have been studied according to the different routes of exposure. Nanoparticles can translocate into the blood and various organs from the respiratory tract and further induce lesions [9]. Many studies have evaluated the toxicity of ZnO NPs in cell lines and animal models [10,11,12,13]. The inhalation of low levels of ZnO NPs causes marked changes and damage to pulmonary function in guinea pigs [14], and ZnO inhalation causes pulmonary impairment and systemic effects such as metal fume fever in humans [15].

Recently, due to the development of biomedical applications, studies for the application of nanomaterials are in progress, and surface modification of nanomaterials is considered essential for application in various fields such as nanocarriers. The physicochemical parameters of nanomaterials have been investigated to affect cellular uptake, etc., and these studies provide important information on the toxicity potential of nanomaterials’ surface modification [16,17,18].

In this study, the used ZnO NP is a nanomaterial discussed as a significant candidate for drug delivery, treatment, and diagnosis [19]. However, the inflammatory response to ZnO NPs in relation to the surface functionalization of ZnO NPs is not clearly understood. Therefore, we used surface-modified (pristine, L-serine, and citrate) ZnO NPs to evaluate the time-point inflammatory responses to respiratory exposure.

## 2. Materials and Methods

### 2.1. Preparation and Characterization of ZnO NPs

The pristine 20 nm sized ZnO NPs were purchased from Sumitomo Osaka Cement Co, Ltd. (Lot number: 141319; Tokyo, Japan). For the pristine ZnO NPs, surface modification was performed to provide surface functionalization according to previously described methods [20,21]. The primary size and morphology of ZnO NPs were measured by scanning electron microscopy (SEM) using Quanta 250 FEG instrument (FEI Co., Hillsboro, OR, USA). The hydrodynamic size and zeta potentials of the ZnO NPs were measured using a Zetasizer Nano ZS (Malvern panalytical, Malvern, UK) in distilled water (DW). Endotoxin was evaluated using an Endpoint Chromogenic Limulus Amoebocyte Lysate assay (Cambrex Corporation, Walkersville, MD, USA).

### 2.2. Animals and ZnO NPs Exposure

Six-week-old specific pathogen-free (SPF) male Wistar rats (200–250 g) were purchased from Samtako (Samtako Bio, Gyeonggi-do, Korea). The animal room was maintained at 20 ± 2 °C, 60 ± 10% relative humidity, and a 12 h light/dark cycle. Distilled water (DW) and sterilized food were available ad libitum to the rats. They were acclimated to this environment for five days prior to dosing. This study was approved by the Institutional Animal Care and Use Committee (IACUC) of the Ministry of Food and Drug Safety (MFDS) (2018, Approval NO. MFDS-18–145). All experimental procedures conformed to the guide and use of laboratory animals. Rats were randomly divided into four groups (*n* = 5 per group): the vehicle (VEH) control group (0.9% saline) and three experimental groups (ZnO NPs modified with pristine, L-serine, and citrate). The experimental groups were intratracheal instilled with single-dose ZnO NPs of 300 µg per rat (volume of 0.5 mL) and evaluated 1 and 30 days after, respectively. Test nanoparticle suspension was prepared and dispersed in 0.9% saline. Then, it was ultrasonically dispersed immediately before administration to animals. The experimental concentration was set by referring to the literature [22]. The control group was treated with 0.9% saline. In the experimental rats, the ZnO NP suspension was administered via intratracheal instillation after they were anesthetized using isoflurane. Instillation of NPs was performed using methods previously described by Lu et al. [21]. At the end of the experiments, the rats were anesthetized by tiletamine-zolazepam (Zoletil^®^) (Virbac, Fort Worth, TX, USA) at 50 mg/kg body weight and sacrificed by exsanguination via the abdominal aorta.

### 2.3. Bronchoalveolar Lavage Fluid Analysis and Cytokine Expression

Bronchoalveolar lavage fluid (BALF) samples were prepared as described previously [23]. BALF was collected by cannulating the trachea and lavaging the lungs three times with 1 mL of cold sterile PBS free of Ca^2+^ and Mg^2+^. Approximately 2 mL of BALF per rat were collected and pooled in sterile centrifuge tubes. The BALF was centrifuged (800× *g* for 10 min at 4 °C) and the total protein was determined in the first 2 mL of BALF by the bicinchoninic acid (BCA) assay (Sigma-Aldrich, St. Louis, MO, USA). Lactate dehydrogenase (LDH) was assayed with a commercial diagnostics kit (Roche Diagnostics Ltd., Burgess Hill, UK). All lavage fluid was pooled and centrifuged at 250× *g* for 10 min at 4 °C. The resulting BALF cell pellet was diluted. Aliquots were analyzed by a hemocytometer and trypan blue dye exclusion for viable cell count and by light microscopy of Diff-Quik-stained (Polysciences) cytospin preparations for cell differentiation. The inflammatory cytokine, interleukin (IL)-4, IL-6, tumor necrosis factor-alpha (TNF-α), and eotaxin in non-diluted BALF were measured using the duoset ELISA kit (R&D System Europe Ltd., Abingdon, UK) and performed according to the manufacture’s procedure. The values of the samples obtained at the two time points (1 day and 30 days) were statistically analyzed by comparing them with the results of the vehicle control group at each time point.

### 2.4. Histopathology

Lungs from the mice of the vehicle control group and treated groups were fixed with 10% neutral buffered formalin and processed according to routine histological techniques. After paraffin embedding, 5 µm sections were cut and stained with hematoxylin and eosin (H & E) for histopathologic evaluation. For the assessment of lung injury, the airways, terminal bronchioles, and lung parenchyma were examined microscopically to evaluate cellular changes and inflammation.

### 2.5. Statistical Analysis

Experimental data were expressed as mean ± standard error of the mean (SEM). The data were analyzed and plotted using GraphPad software (ver. 5; GraphPad Software Inc., La Jolla, CA, USA). All results were compared by one-way ANOVA with post hoc Tukey’s pair-wise analysis. *p* values of less than 5% (*p* < 0.05) were considered statistically significant.

## 3. Results

### 3.1. Physicochemical Properties of ZnO NPs

**Figure 1:**
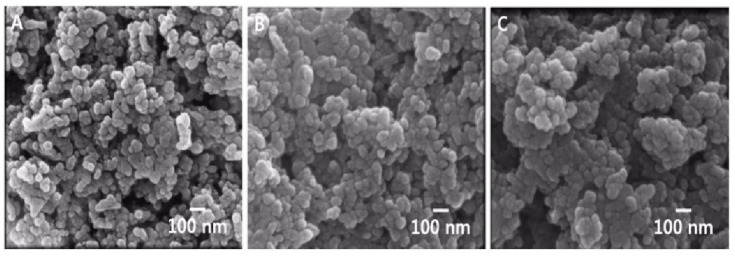
Scanning electron microscopy (SEM) image of three types of ZnO NPs. ZnO NPs were deposited on SEM grids and directly observed. SEM image showing that ZnO NPs were uniformly spherical (**A**) 20 nm ZnO NPs (pristine), (**B**) 20 nm ZnO NPs (L-serine), and (**C**) 20 nm ZnO NPs (citrate). (Scale bar = 100 nm). The information on surface-modified 20 nm ZnO nanoparticles was taken from the literature previously reported by Yang et al. [20].

The physicochemical properties of ZnO NPs are summarized in Table 1. To evaluate the colloidal properties and behavior of ZnO NPs in aqueous systems, we investigated the particle size and morphology of ZnO NPs dispersed in deionized water. The three types of ZnO nanoparticles with a primary size of approximately 26–27 nm were shown to aggregate in water to a size of 219–399 nm. In addition, the SEM image showed agglomerated morphology between particles, including spherical features (Figure 1). As for the zeta potentials, the pristine group showed a charge of approximately 21 mV, ZnO with an L-serine group attached to it showed a charge of about 30 mV, and ZnO with a citrate group had a charge of −36 mV. The contamination by endotoxin was not observed for all substances.

### 3.2. Bronchoalveolar Lavage Fluid Analysis

**Figure 2:**
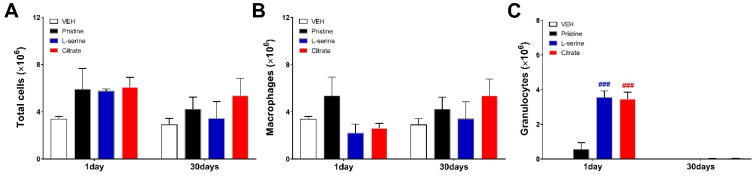
Differential cells in bronchoalveolar lavage fluid (BALF) from rat lungs at days 1 and 30 after a single instillation of ZnO NP groups. Number of (**A**) total cells, (**B**) macrophages, and (**C**) granulocytes. Data are expressed mean ± standard error of the mean (SEM) (*n* = 5). Significance versus vehicle (VEH) control: ### *p* value < 0.0001.

**Figure 3:**
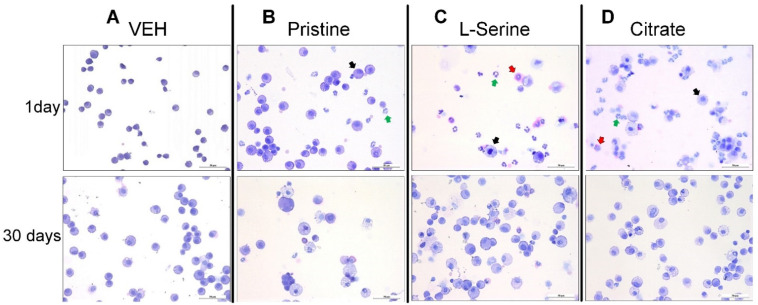
Morphology of differential cells in bronchoalveolar lavage fluid (BALF) from rat lungs at days 1 and 30 after a single instillation of ZnO NP groups. Representative BALF cell images (cytospin preparation stained with Diff-Quik) after intratracheal instillation of ZnO NPs groups: (**A**) vehicle control (VEH); and ZnO NPs groups of (**B**) pristine, (**C**) L-serine, and (**D**) citrate. Pictures were taken at ×200 magnification (scale bar = 50 μm, red arrows = eosinophil, green arrows = neutrophil, and black arrows = macrophage).

To evaluate the inflammatory pattern of differential ZnO NPs over time, a cytological analysis of BALF was performed (Figure 2 and Figure 3). Then, 1 and 30 days after ZnO NP injection, the total cells of BALF were higher than those of vehicles in all three groups (Figure 2A). The ZnO group with L-Serine (positive charge induction) and citrate (positive charge induction) functional groups induced a significant increase in granulocytes on day 1 and recovered to the control level after 30 days (Figure 2C). The pristine group induced relatively lower granulocytes than the surface-functionalized L-serine and citrate groups, and macrophages and neutrophils were observed mainly in BALF (Figure 2B and Figure 3B).

### 3.3. Changes in Biochemical Indicators following Instillation with ZnO NPs

**Figure 4:**
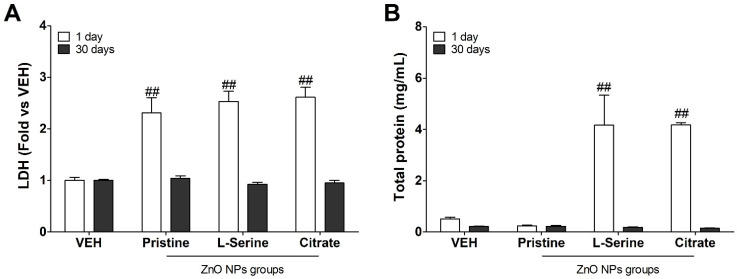
Lactate dehydrogenase (LDH) and total protein in rat lungs following intratracheal instillation of differential functionalized ZnO NPs. Level of (**A**) LDH and (**B**) total protein from 1 to 30 days in bronchoalveolar lavage fluid (BALF). Data are expressed mean ± standard error of the mean (SEM) (*n* = 5). Significance versus vehicle (VEH) control: ## *p* value < 0.001.

The LDH enzymes are distributed widely in the cytosol during the conversion of lactate to pyruvate. The NP’s cytotoxicity might break the plasma membrane integrity, causing LDH to leak into BALF, with an increase in extracellular levels. The level of LDH was highest on the first day after administration (Figure 4A). The total protein, a marker for vascular permeability, significantly increased at day 1 in the functionalized (L-serine and citrate) ZnO NPs groups compared with the vehicle control and pristine group (Figure 4B). On day 30 after instillation, the levels of LDH and total protein in all groups recovered to the control level.

### 3.4. Inflammatory Cytokine Expression following Instilled with ZnO NPs

**Figure 5:**
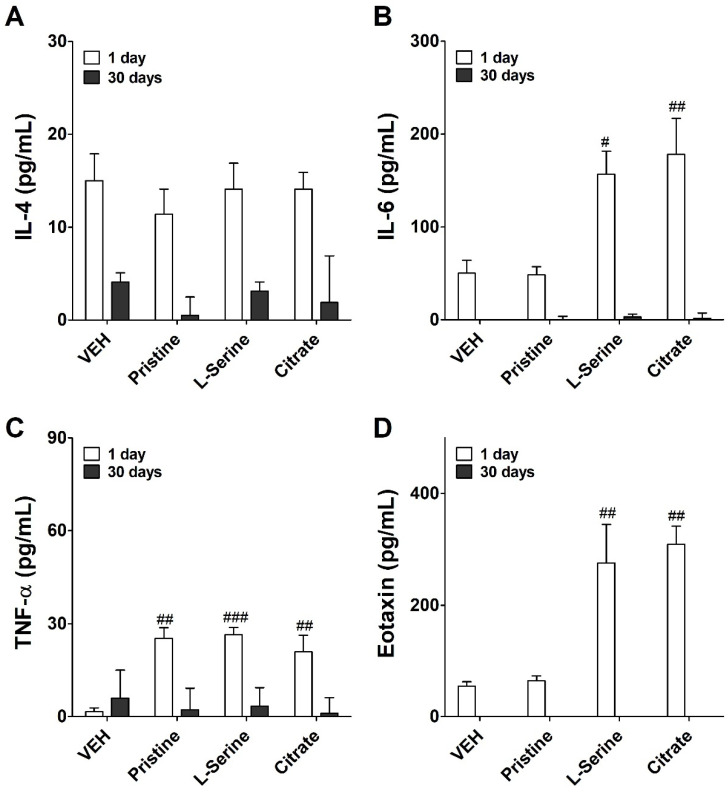
Evaluation of inflammatory cytokines cells in bronchoalveolar lavage fluid (BALF) from rat lungs at day 1 and 30 after a single instillation of ZnO NPs. Level of (**A**) interleukin (IL)-4, (**B**) IL-6, (**C**) tumor necrosis factor (TNF) −α, and (**D**) eotaxin. Data are expressed means ± standard error of the mean (SEM) (*n* = 5). Significance versus vehicle (VEH) control: # *p* value < 0.05, ## *p* value < 0.001, and ### *p* value < 0.0001.

To identify inflammatory responses induced by the instillation of differential ZnO NP groups, the levels of inflammatory cytokines were measured in the BALF (Figure 5). IL-4 is a cytokine that induces the differentiation of helper T cells into Th2 cells. The level of IL-4 showed a control or lower value on day 1 and day 30 (Figure 5A). IL-6 can act as both pro- and anti-inflammatory cytokines, and levels of IL-6 increased significantly on day 1 in the ZnO NPs-instilled group functionalized with L-serine and citrate rather than the pristine group. Then, it decreased sharply on day 30 after intratracheal instillation (Figure 5B). TNF-α is a cell-signaling cytokine involved in systemic inflammation and one of the cytokines responsible for causing the acute phase reaction. Regardless of the functional group, TNF-α levels were significantly increased at day 1 in the ZnO NPs-instilled groups, decreasing by day 30 after the instillation (Figure 5C). Eotaxin is a CC chemokine subfamily of eosinophil chemotactic proteins, and functionalized ZnO NPs groups significantly increased the level of eotaxin on day 1 rather than the pristine group (Figure 5D). Eotaxin also recovered to the control level on day 30.

### 3.5. Histopathology

**Figure 6:**
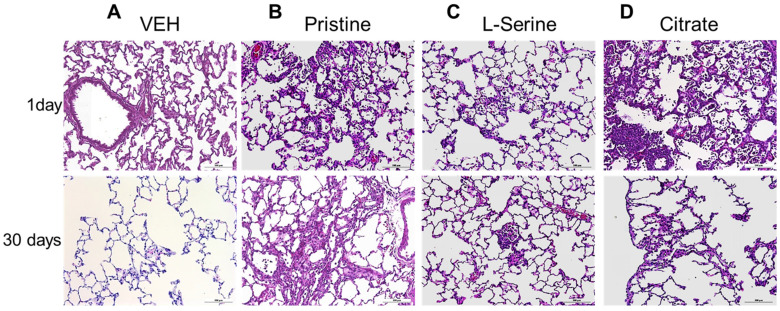
Lung histopathology of rat at day 1 and 30 after a single instillation of ZnO NPs. Representative lung images (stained with H & E) after intratracheal instillation of 20 nm ZnO NPs groups: (**A**) vehicle (VEH) control, (**B**) pristine, (**C**) L-serine, and (**D**) citrate ZnO NPs group. Day 1, infiltration of granulocytes in alveoli was observed (**B**–**D**, 1 day). Day 30, fibrosis in alveolar wall was observed (**B**–**D**, 30 days). Pictures were taken at ×200 magnification (Scale bars = 50 μm).

Figure 6 presented histological lesions at 24 h and 30 days of single exposure with a group of functionalized ZnO NPs. In acute inflammation 24 h after instillation of all ZnO NP groups, inflammatory cells including neutrophils infiltrate the alveolar and bronchoalveolar junction. Infiltration of inflammatory cells led to an increase in the alveolar wall thickness. This was consistent with BALF cell analysis data (Figure 2 and Figure 6). For example, in all ZnO groups, the increase in alveolar granulocytes increased on day 1 and recovered on day 30, regardless of functional group, and a recovery pattern accompanied by fibrosis was observed in lung tissue as it also progressed from day 1 to day 30. However, in contrast, the pristine group, which had relatively few granulocytes in the BALF cell analysis, clearly showed high granulocyte penetration in the alveolar and bronchoalveolar junction.

## 4. Discussion

The US National Nanotechnology Initiative defines nanomaterials as materials with at least one dimension in the range of 1–100 nm [24]. Significant increases in production and demand could lead to unintended exposures to nanomaterials not only by workers in certain industries, but also by end-product users via inhalation, dermal absorption, and gastrointestinal tract absorption [25]. Because of their unique properties, including small size and a corresponding large specific surface area, nanomaterials are believed to impose different degrees of biological effects from those of microscale materials [26]. Inhalation studies show that, in comparison with their larger-sized counterparts, nanoparticles penetrate deeper into the lungs and become localized within various cell types, inducing a more significant inflammatory response associated with marked potential toxicity [7,27,28]. 

ZnO NPs are representative nanomaterials that can be used in biomedical applications such as biosensors. For example, ZnO NPs or ZnO composites are applied for vaccine-adjuvant use because the form of NPs can efficiently present antigens to dendritic cells (DCs) with subsequent adequate inflammation [29,30]. However, many studies have been reported on the inhalation toxicity of ZnO NPs, but little information is available on the effects of surface-modified ZnO NPs exposure to the lungs.

The ZnO NPs synthesized with three different functional groups (pristine, L-serine, and citrate) were used in the current study, as described in the previously reported literature [20]. Since a total of three types of NPs groups share the same core (ZnO NPs), it was confirmed that they have very similar primary sizes and shapes. By the functional groups of the NPs, ZnO containing L-serine exhibited a positive charge, and ZnO containing citrate exhibited a negative charge. However, we could not find any evidence of a specific toxicity correlation according to the differential surface charge of the synthesized ZnO NPs. The presence of endotoxin can lead to inflammation of the lungs, resulting in distortion of the test substance’s results [31]. We evaluated the endotoxin contamination of the test substance before the test, removing the influence of the endotoxin impurity of the test substance and securing the reliability of the result.

Intratracheal injection of functionalized (L-serine and citrate) ZnO NPs significantly increased the total protein in BALF on day 1, consistent with the BALF cytometric results. The increase in total protein, an indicator of vascular permeability of inflammatory cells, promoted the migration of granulocytes from blood vessels to tissues [32]. In the case of pristine ZnO NPs, the BALF analysis results showed that granulocytes were recruited higher than those of the control group. Still, inflammation by macrophages appeared to be mainly on day 30 after instillation, when inflammatory cells of macrophages and granulocytes decreased, and the levels of LDH and total protein, which are cytotoxic indicators, were also decreased. As reported, ZnO nanoparticles induced acute (24 h) inflammation, and a similar trend was observed to recover over time (30 days) [22]. As can be seen through the rat lung at 30 days after administration, the number of inflammatory cells clearly decreased in the alveoli. Beyond the presentation of some localized inflammation, fibrosis and recovery were observed as well.

The results of inflammatory cytokine analysis in BALF showed that the cytokine levels in rats treated with all ZnO NPs were generally increased compared to the control group. Of these cytokines, IL-6 and TNF-α were prominently affected by ZnO NPs treatment. These cytokines are representative pro- and anti-inflammatory cytokines, respectively. IL-6 acts as a pleiotropic cytokine, with important biological effects on inflammation, immunity, and stress [33]. Researchers have confirmed the ability of ZnO NPs to induce TNF-α or IL-6 production in purified primary cell cultures or toxicological evaluation [10]. It is noteworthy that the inhalation of ultrafine ZnO NPs in occupational settings can increase the expression of these cytokines, which is symptomatically recognized as metal fume fever in welders [34]. The genes of these IL-6 play pivotal roles in the inflammation induced by ZnO NPs when the treatment doses are high. In this study, as a result of cytokine analysis of the pristine group (Figure 5C), an inflammatory response by macrophages induced an increase in TNF-alpha. On the other hand, the induction of IL-6 cytokines was not made. As a result, it is estimated that the recruitment of granulocytes is remarkably low because pristine ZnO nanoparticles do not induce IL-6 (Figure 2C and Figure 5B). Another study using IL-6 gene silencing showed that, in induced lung fibrosis, IL-6 played an important role in the regulation of the expression of Th2 cytokines (IL-4 and IL-5) [35]. However, in our study, the induction of IL-4, a Th2-related cytokine, was very insignificant.

Although it cannot be confirmed in the current study, it is possible that other pathways promoted the increase in eotaxin. In addition, the IL-6 receptor regulates fibrosis in tissues by regulating the profibrotic cytokine [35,36,37]. The ability of ZnO NPs to induce TNF-α may also help regulate acute inflammation and promote Th1 differentiation [22]. As another role, it has been reported that the TNF-α can induce the accumulation of eotaxin mRNA in lung epithelial cell lines A549 and BEAS 2B in a dose-dependent manner [38]. Further research will be needed, but it is assumed that the increased levels of eotaxin in our study were affected by the TNF-alpha cytokine. Eosinophils play a key role in mediating asthma and other allergic conditions, and ZnO NPs can induce eosinophilic to severely cytotoxic inflammation [38]. Eotaxin is a chemokine that plays a vital role in recruiting eosinophils. Moreover, as previously reported, lung exposure of ZnO NPs has been shown to induce eosinophil-related inflammation at 24 h [22].

In this study, tests were conducted with two limited functional groups, L-serine and citrate, but more diverse functional groups can be attached to the surface of actual nanomaterials [39,40]. In the field of biomedical engineering, there is an active movement to utilize surface-functionalized nanomaterials; thus, in order to secure the safety of nanomaterials modified with functional groups, it is necessary to accumulate toxicity data through more studies.

The intratracheal instillation method used in this study has the advantage of being able to inject a solution with a precisely quantified concentration into the lungs. However, in actual exposure cases, individual subjects may not be inhaled at exactly the same amount, so it is necessary to establish an endpoint of toxicity through multi-disciplinary studies.

## 5. Conclusions

The results found in this study indicate that exposed ZnO NPs to the lungs induced potent but reversible inflammation in the acute phase, which recovered 30 days after instillation. In addition, although no obvious difference was observed according to the functional groups, it suggests that when L-serine and citrate functional groups are attached to the core, the production of IL-6 and eotaxin can be increased, thereby promoting the recruitment of granulocytes.

## Figures and Tables

**Table 1:** Physicochemical properties of three types of ZnO NPs.

Modification		20 nm ZnO NPs	
Measurements	Pristine	L-Serine	Citrate
Primary size (nm) ^a^	26.8 ± 4.5	27.1 ± 7.5	26.9 ± 4.3
Hydrodynamic size (nm) ^b^	399 ± 16	219 ± 3	341 ± 61
Polydispersity (PDI) ^b^	0.50 ± 0.02	0.62 ± 0.07	0.63 ± 0.06
Zeta potential (mV) ^b^	21.4 ± 4.6	30.0 ± 3.4	−36.0 ± 2.6
Endotoxin (Unit/mL)	<0.1	<0.1	<0.1

^a^ Primary size was measured by scanning electron microscopy (SEM) data. ^b^ Hydrodynamic size, polydispersity, and zeta potential were measured in distilled water (DW). The information on surface-modified 20 nm ZnO nanoparticles was taken from the literature previously reported by Yang et al. [20].

## Data Availability

The original contributions presented in the study are included in the article further inquiries can be directed to the corresponding authors.
